# Behavioral Economics and Innovation Uptake: Building New Capabilities to Overcome Barriers to Technology-Enabled Care

**DOI:** 10.5935/abc.20190216

**Published:** 2019-10

**Authors:** Alexandre Siciliano Colafranceschi

**Affiliations:** 1 Universidade Federal do Estado do Rio de Janeiro, Rio de Janeiro, RJ - Brazil; 2 Instituto Nacional de Cardiologia, Rio de Janeiro, RJ - Brazil; 3 Hospital Pró-Cardíaco, Rio de Janeiro, RJ - Brazil

**Keywords:** Telemedicine/economics, Telemedicine/legislation and jurisprudence, Telemedicine/trends, National Health Science, Technology and Innovation Policy, Telemedicine/methods

## Introduction

The Federal Medical Council (CFM) has recently published a new resolution on telemedicine in Brazil. The 2227/2018 resolution, establishing the criteria for the use of telemedicine, was published in *Diario Oficial da União* (DOU, the official journal of the federal government of Brazil) on February 6, 2019.^1^ This new policy, aimed at defining telemedicine as a way of providing medical services by means of technology, is much more aggressive than the previous one published in 2002, which limited the use of telemedicine to medical consultations made by telephone or internet, and implied the presence of a health professional at both ends of the communication channel. The current resolution expanded the concept of telemedicine in providing technological solutions for remote patient monitoring and treatment (drug prescription and surgical interventions), and analysis of laboratory results. However, soon after its release, the new document caused an intense public debate on the theme dividing stakeholders for and against the incorporation of telemedicine into practice nationwide. The debate was so intense that the Federal Medical Council revoked the resolution, as published in the DOU on March 6, 2019.^2^

The revocation of the policy after intense societal debate indicates the significant challenges regarding the implementation of technology-enabled care in Brazil. Such debate should not be the end, but rather, the beginning of a social mobilization to reframe the use of connected technology in healthcare in the country. To tackle the barriers to the uptake of technology-enabled care in Brazil, one should better understand the stakeholder positions, and consider the political and cultural environment, the ethical and legal apparatus and the available technology infrastructure.^3^

A more comprehensive understanding of the contemporary scenario where technology-enabled care may fit into Brazilians’ needs is critical to suggest approaches that might lead to societal benefit while being acceptable by physicians and other stakeholders.

## Objectives

This is an exploratory paper, aiming to provide a personal overview of potential barriers to the incorporation and dissemination of telemedicine in Brazil. It is also an objective to discuss potential approaches to overcome these barriers.

### A) Analysis of the stakeholders

#### A.1) The State and Telemedicine - The Government as a stakeholder

From the political point of view, government initiatives concerning telemedicine have been led primarily by the Brazilian Ministry of Health and were designed to promote the use of telemedicine in the expansion and improvement of health services. The dimensions involved, however, are beyond the limits established by the Ministry of Health. An effective inter-ministerial action would be required to foster the economy (innovation and economic efficiency) and the social dimension (interest of the population and equality) to leverage telemedicine towards the expansion and improvement of healthcare.

#### A.2) Challenging the “status quo” - Providers as stakeholders: institutions, physicians and other practitioners

Culture is another limiting factor in the dissemination of telemedicine from the perspective of institutions, physicians and other practitioners. From the need for adjusting to the new working process, to the challenging relationship between power structure and professional structure, the adoption of the latest technology may generate significant resistance. The resistance to change is strengthened by the risk aversion^4^ and uncertainties that are commonly related to the introduction of a "new way of doing things". On the one hand, technology-enabled care may help overcome the obstacle of access posed by the distance (especially in a continental-dimension country like Brazil) with expected gains with the information and communication technologies, *i.e*., by increasing access and reducing costs. However, the interdependence between telemedicine and health services organization in guiding new investments may cause a shift in the arena of power. These complexities and uncertainties pose a substantial barrier to the dissemination of the new technologies.

Likewise, telemedicine faces resistances from practitioners.^2^ Telemedicine involves multidisciplinary players, encompassing health practitioners of diverse disciplines, information and communication technology experts, managers and policy makers. The adoption of this technology requires the redesigning of work processes in their multiple aspects that generate tensions and conflicts. Physicians, in general, are not trained to be part of a “real team" and tend to behave more like a chairperson, which can increase the tension among team members. Moreover, telemedicine changes the typical doctor-patient relationship, requiring a process of acceptance, by all, of the technological mediation. Beyond that, physicians believe that these technologies may constitute an unsafe medical practice, in part due to the infeasibility in performing a remote physical examination. Overcoming institutional and professional cultural barriers is an essential step in the process of telemedicine dissemination and consolidation. Finally, reimbursement is another issue; physicians feel they will be pressured to care for more patients, dedicating less time to each patient, with lower reimbursement rates.

#### A.3) Patients: what do they want? Are they willing to trade off? Consumers as stakeholders

From the patient perspective, although telemedicine may add distinct value to their needs and cheaper access to healthcare, as a health consumer, they may fail to buy the "innovative product" because it may require them to change their behavior.^5^ Although it may be cheaper in financial terms, there are psychological costs associated with behavior changes: people irrationally overvalue benefits they currently possess relative to those they do not.^6^

### B) Behavioral economics and innovation uptake

The understanding of the psychology of gains and loss, and more deeply, the concepts of loss aversion, *status quo* bias, and the endowment effect,^4,6^ associated with why the adoption of innovation fails^5^ may help propose specific solutions where telemedicine may be acceptable by providers and wanted by patients.^7^ Examples of approaches regarding telemedicine that might lead to societal benefit while being fair to physicians are described below:

**B.1) To make behaviorally compatible products:** the development and incorporation of mobile health sensors may offer a sense of safety that is missing to remote physicians. If one can rely on such type of device for feedback of a "remote” physical examination, physicians would feel more secure in guiding and discussing about a patient’s condition using technology mediation. This may minimize physicians’ resistance to telemedicine.

**B.2) Seek out the deprived individuals (the ones with no access to healthcare):** telemedicine has the potential of solving significant current health challenges. In addition to the Brazilian territorial extension, there are thousands of isolated, difficult-to-access locations where healthcare services are extremely scarce, and physicians are lacking. Some physicians are mandated to serve in remote areas (military physicians). Fostering the development of the required infrastructure for the establishment of telemedicine in remote areas will open the doors for communities to have access not only to healthcare but also to other resources (like education). This will promote secondary gains as enhancing local and regional economic progress and may attract physicians and their families to places that otherwise would not be the first choice to live.

**B.3) Find believers (Millennials):** According to Ripton,^8^ millennial generation has been changing healthcare by forcing a greater emphasis on technology solutions for healthcare delivery. The development of technology-enabled solutions targeting this population may speed up and sustain the adoption of telemedicine not only in Brazil but also in other countries. The millennials’ demand will force physicians to adapt (and incorporate) to technology-enabled care, to be competitive in the market.

**B.4) Strive for 10x improvements**^5^**:** telemedicine benefits should be so great that it would overcome physicians and patients’ overweighing of potential losses. Besides adding efficiency and reducing costs, telemedicine has the potential to expand the actions of health practitioners, integrating them into healthcare services and systems. Also, one can explore the potential savings and share them with practitioners in a new type of employment relationship and reimbursement model that may improve acceptance of telemedicine among physicians while promoting societal benefits.

### C) Other considerations

#### C.1) Ethics and legal issues in the digital age: is technology changing faster than expected?

Besides what has been discussed above, there is also a lack of synchronization between the vast potential of these technologies and the prevailing ethical and legal apparatus. Contrary to a comprehensive national policy, there is a general scenario of fragmentation, characterized by different norms and standards issued by various bodies and with distinct focuses.^3^ Even though a single instrument would hardly reach these goals, the fragmentation is one more hurdle to overcome to achieve the potential of telemedicine.

#### C.2) Infrastructure - Are humans slower than expected?

Also, mention should be made of the scarcity of resources and technical expertise, as well as infrastructure issues. Brazil has unequal geographic distribution concerning broadband availability.^3^ This means the infrastructure of the broadband data network is one of the most limiting factors to the expansion of telemedicine, particularly, in the countryside of Brazil.

#### C.3) Health services in Brazil

Finally, it should be mentioned the precariousness of health services in Brazil, including primary care facilities, outpatient clinics, and even specialized hospital services. Scarce resources, management problems, lack of practitioners, inadequate compensation, outdated facilities, lack of equipment and consumables, among many other aspects, are repeatedly mentioned as the leading causes of such precariousness, witnessed by health professionals and users. This is even worse in remote and peripheral areas and is a significant barrier to the dissemination and consolidation of telemedicine in Brazil.^3^ Therefore, even with the implementation of all technological infrastructure required for telemedicine, which is typically an interdisciplinary activity, it would not guarantee an improved and more expanded access to healthcare.

## Conclusion

The primary characteristic of telemedicine is its ability to make access to health services democratic. To accomplish that, legislative initiatives (economic and social) that support and encourage the use of this technology, a regulatory apparatus, mobilization of a core group of companies, and development of scientific capability are required. From the healthcare perspective, telemedicine is capable of promoting higher integration of the healthcare system, overcoming the still existing and deleterious fragmentation that prevents the access to full healthcare rights. Investments in infrastructure are mandatory to widespread adoption of telemedicine. Beyond that, other challenges that limit its development are most related to the stakeholder’s conflicts, interdependence, and demands. In this regard, understanding some of the concepts related to behavioral economy and innovation uptake failure may increase or create opportunities and approaches where the use of technology-enabled care might lead to societal benefit while being acceptable to physicians and other stakeholders.
